# Supplementary addendum to “Non-radiographic methods of measuring global sagittal balance: a systematic review”; Reliability of the Spinal Mouse in adult back pain sufferers

**DOI:** 10.1186/s13013-018-0167-x

**Published:** 2018-09-02

**Authors:** Larry Cohen, Sarah Kobayashi, Milena Simic, Sarah Dennis, Kathryn Refshauge, Evangelos Pappas

**Affiliations:** Faculty of Health Sciences, Discipline of Physiotherapy, The University of Sydney, 75 East Street, Lidcombe, NSW 2141 Australia

We would like to provide an update for the paper “Non-radiographic methods of measuring global sagittal balance: a systematic review” in *Scoliosis and Spinal Disorders* [1] with additional data regarding the reliability of the Spinal Mouse method in evaluating global sagittal balance through trunk inclination in an adult population with back pain.

We were alerted to the presence of additional data [2] fitting the inclusion criteria documenting excellent intra-rater reliability (ICC 0.845 [CI 0.679–0.925], SEM 0.803°) of the Spinal Mouse system in a population of 50 adults with back pain aged 58.4 ± 13.4 years. This reliability data is consistent with the reported results in healthy children and healthy adults.

Tables 1 and 2 are updated as below.

**Table 1:** Methodological quality of included studies evaluated using the Brink and Louw critical appraisal tool

Key information	1	2	3	4	5	6	7	8	9	10	11	12	13	High quality > 60%
Topalidou et al. 2014	✓	✘	n/a	n/a	✘	n/a	n/a	✓	n/a	✓	n/a	✘	✓	4/7 = 57%

Item key: 1—description of study population, 2—description of raters, 3—explanation of reference standards (validity only) 4—between rater blinding (reliability only), 5—within rater blinding (reliability), 6—variation of testing order (reliability), 7—time period between index test and reference standard (validity), 8—time period between repeated measures (reliability), 9—independency of reference standard from index test (validity), 10—description of index test procedure, 11—description of reference test procedure (validity), 12—explanation of any withdrawals, 13—appropriate statistics methods. Legend: ✓ reported, ✘ not reported

**Table 2:** Study characteristics, reliability, validity and SEM data of included studies

Non-radiographic method	Study	Index test variable	Sample	Age	Methodology description	Reliability test variable	Statistical measure	Statistical value	SEM
Spinal Mouse	Topalidou et al. 2014	C7-S1 Angular trunk inclination	50 adults with back pain.	58.4 ± 13.4 years	Examined by1 rater on 2 separate occasions, 30 min apart	Intra-rater	ICC	0.845	0.8°

*SEM* standard error of measurement
